# Impact of beneficiary status on bariatric surgery outcomes in a military treatment facility

**DOI:** 10.1007/s00464-026-12860-z

**Published:** 2026-05-18

**Authors:** Michael T. Olson, Yun Beom Lee, Pamela Masella, Brian D. Layton

**Affiliations:** Department of General Surgery, Brooke Army Medical Center, 3551 Roger Brooke Drive, San Antonio, TX USA

**Keywords:** Bariatric surgery, Beneficiary, Dependent, Healthcare utilization, Retiree

## Abstract

**Background:**

Retirees and dependents represent demographically and clinically distinct beneficiary groups within the Military Health System (MHS). We assessed whether beneficiary status influences perioperative outcomes, healthcare utilization, and weight loss after bariatric surgery.

**Methods:**

We conducted a retrospective cohort study of adults undergoing sleeve gastrectomy or Roux-en-Y gastric bypass from 01/01/2022 to 12/31/2024 at a single military treatment facility. Outcomes were stratified by beneficiary status (retiree versus dependent). Propensity scores incorporating demographic, clinical, and procedural factors were used to construct overlap weights, yielding covariate balance comparable to randomized allocation. Overlap-weighted logistic and linear regression models assessed the independent association between beneficiary status and postoperative outcomes. Sex interaction terms evaluated effect modification. A female-only 1:1 propensity score-matched analysis served as a sensitivity analysis to address marked sex imbalance between groups.

**Results:**

Among 281 patients, 123 (43.8%) were retirees and 158 (56.2%) dependents. Retirees were older (median 50 versus 46 years, *p* = 0.003), predominantly male (52% versus 7%, *p* < 0.001), higher frequency Black (12.2% versus 5.1%, *p* = 0.052) and Hispanic (22.8% versus 11.4%, *p* = 0.017) and had higher comorbidity burden (median count 5.0 versus 4.0, *p* < 0.001). Revision/conversion procedures were less common in retirees; otherwise, operative indices were comparable. In unadjusted analyses, retirees had shorter hospital length of stay (LOS), fewer all-cause ED visits at 30 days, and fewer surgical ED visits through 12 months. However, in weighted regression, beneficiary status was not independently associated with LOS, ED visits, readmissions, or short-term percent total weight loss. No significant sex–beneficiary status interaction was observed. In the female-only matched cohort (*N* = 114; 57 pairs), outcomes again remained comparable.

**Conclusions:**

After covariate adjustment using overlap weighting and sex-restricted propensity matching, beneficiary status was not independently associated with LOS, postoperative healthcare utilization, or short-term weight loss. These findings suggest equitable bariatric outcomes across MHS beneficiary groups.

**Graphical abstract:**

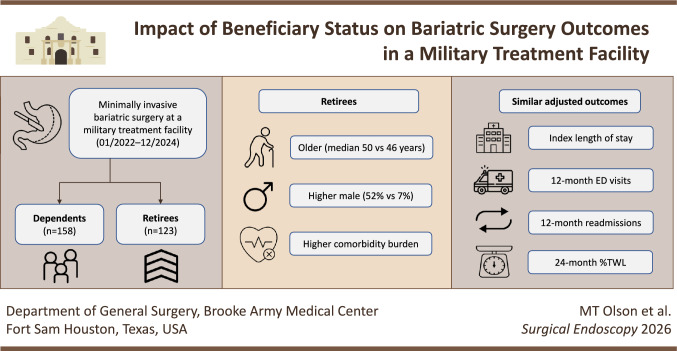

**Supplementary Information:**

The online version contains supplementary material available at 10.1007/s00464-026-12860-z.

The Military Health System (MHS) delivers healthcare to a heterogeneous population that includes active-duty service members, retirees, and dependents. Retirees and dependents represent distinct beneficiary subgroups, differing substantially in age distribution, medical comorbidity burden, stress-related behaviors, and anthropometric meaures [1–3]. Across medical and surgical fields, beneficiary status within military systems has been associated with differences in healthcare access, chronic disease prevalence, and rates of receiving select procedures, even bariatric surgical procedures [4, 5]. However, the relationship between beneficiary category and outcomes after bariatric surgery has not been rigorously examined.

Bariatric surgery is the most effective intervention for obesity and metabolic disease [6]. Understanding whether access or outcomes differ between retiree and dependent populations receiving bariatric surgery in the MHS has implications for resource allocation, equitable care delivery, and long-term health system planning. Prior literature in the civilian sector suggests that socioeconomic status, insurance type, and demographic factors influence postoperative outcomes including readmissions, emergency department (ED) utilization, and long-term weight loss [7–9]. Whether these relationships extend to the unique MHS beneficiary structure remains unclear.

Very limited prior work from previous colleagues at our institution has evaluated clinical outcomes among military beneficiaries undergoing bariatric surgery [10]. A small series with their preliminary results found notable differences in comorbidity profile between retirees and dependents, as well as lower short-term weight loss in retirees. Nonetheless, no comprehensive evaluation of perioperative outcomes, postoperative healthcare utilization, and weight loss durability has been conducted. Notably, retirees undergoing bariatric surgery are substantially more likely to be male and older [11], both of which are factors independently associated with postoperative risk and variability in postoperative outcomes [12]. Therefore, any assessment of beneficiary status must carefully adjust for demographic imbalance, particularly sex, to avoid conflating beneficiary category with unbalanced sex distributions.

The objectives of this study were to compare perioperative outcomes, postoperative healthcare utilization, and longitudinal metabolic and weight loss outcomes between retirees and dependents undergoing bariatric surgery, and to determine whether beneficiary status is independently associated with these outcomes after accounting for key demographic and clinical differences. By addressing these questions, we sought to clarify whether beneficiary category represents a meaningful determinant of bariatric surgery outcomes.

## Materials and methods

### Study design and setting

This was a retrospective, single-center cohort study conducted at a United States military treatment facility (MTF). Institutional review board approval was obtained with waiver of informed consent.

### Patient selection and grouping

The study included all adult patients (≥ 18 years) who underwent bariatric surgery between January 1, 2022 and December 31, 2024, including both primary and revisional (and conversion) minimally invasive sleeve gastrectomy (SG) and Roux-en-Y gastric bypass (RYGB). All procedures were performed by two fellowship-trained bariatric surgeons. Exclusion criteria included other bariatric-related cases performed by both surgeons in the same time period, including isolated adjustable gastric band removals, bariatric reversal procedures, remnant gastrectomy, or procedures performed for bariatric complications, such as fistula takedown. All patients were managed according to the same standardized Enhanced Recovery After Surgery protocol for bariatric procedures. Patients were grouped as retiree versus dependent based on Defense Enrollment Eligibility Reporting System classification. Active duty service members were not included in this analysis, as current military policy considers bariatric surgery a disqualifying condition for continued service due to required long-term dietary and lifestyle modifications that may limit deployability. As such, bariatric procedures were not performed in active-duty personnel within this setting.

### Data collection and outcomes

Demographic variables included age, sex, race, marital status, and distance from home to our institution. Clinical variables included preoperative weight, height, body mass index (BMI), ideal body weight, excess body weight, comorbidity burden, relevant medication use, and laboratory and metabolic parameters, including hemoglobin, hemoglobin A1c (HbA1c), and lipid profile. Operative characteristics included procedure type, operative approach, operative time, and intraoperative complications. Perioperative characteristics included length of stay (LOS), opioid and antiemetic use, oral fluid intake, and postoperative complication rate.

Primary outcome was postoperative healthcare utilization at 12 months, which included all-cause and surgical ED visits and readmissions. A ‘surgical’ ED visit was defined as any ED visit where a surgical consult was required, which was verified by documentation from the on-call bariatric surgery service. Secondary outcomes included index hospital LOS, postoperative complications and reoperations at 12 months, laboratory and metabolic changes at 12 months, and weight loss (percentage total weight loss [TWL] and excess weight loss [EWL]) at 6, 12, and 24 months.

### Rationale and statistical analysis

Continuous variables were compared using Student’s *t*-tests or Wilcoxon rank-sum tests, and categorical variables were analyzed using *χ*^*2*^ or Fisher’s exact tests. Because retirees and dependents differed markedly at baseline, particularly in sex distribution, unadjusted comparisons were expected to be confounded. To estimate the independent association between beneficiary status and outcomes, we used causal adjustment with propensity-based methods.

Propensity scores for retiree versus dependent status were generated using logistic regression including age, sex, BMI, major comorbidities, procedure type, and revisional status. Overlap weighting was selected as the primary analytic approach because it retains all patients, minimizes the influence of extreme propensity scores, and yields covariate balance similar to randomized allocation. Balance before and after weighting was assessed using standardized mean differences and Love plots. Weighted logistic and linear regression models were then used to evaluate perioperative outcomes, healthcare utilization at 30 days, 6 months, and 12 months, and weight loss at 6, 12, and 24 months. Results were reported as odds ratios (OR) or beta coefficients (*β*) with 95% confidence intervals. Marginal-effects analyses were used to generate adjusted predicted probabilities and means.

Because of the marked sex imbalance between beneficiary groups, we incorporated beneficiary status × sex interaction terms into weighted models to assess potential effect modification. To validate findings in a sex-balanced cohort, we performed a secondary female-only propensity score matching analysis using 1:1 nearest-neighbor matching without replacement (caliper 0.2 SD). Covariate balance in the matched cohort was reevaluated using standardized mean differences and Love plots. Outcomes in the matched cohort were analyzed using regression models with the same covariates used in the weighted analyses.

Lastly, as this was a retrospective fixed-cohort analysis, an a priori sample size calculation was not performed. To inform interpretation of non-significant results, we conducted a post hoc detectable-difference assessment for 12-month all-cause ED utilization and readmission, estimating the minimum absolute risk difference detectable with 80% power at a two-sided *α* of 0.05. Overall, *p* values < 0.05 were considered significant. All analyses were performed in R version 4.3.3 (R Foundation for Statistical Computing, Vienna, Austria).

### Reporting standards

This study was designed, conducted, and reported in accordance with the Strengthening the Reporting of Observational Studies in Epidemiology (STROBE) guidelines for observational research [13]. 

## Results

### Overall cohort characteristics

A total of 293 patients underwent bariatric surgery at our institution during the study period. Twelve patients were excluded based on procedure type: adjustable gastric band removal (*N* = 7), bariatric reversal procedure (*N* = 2), remnant gastrectomy (*N* = 1), and gastrogastric fistula repair (*N* = 2). A total of 281 patients were included in the study, grouped into 123 retirees (43.8%) and 158 dependents (56.2%). The overall cohort had a median age of 48.0 (41.0–55.0) years and was predominantly female (73.3%). Roux-en-Y gastric bypass was the most common procedure performed overall (55.9%). Median hospital LOS was 1.0 (1.0–2.0) days.

### Unadjusted comparisons between beneficiary groups

#### Demographics and preoperative characteristics

Table 1 shows the demographic and preoperative characteristics stratified by beneficiary group. Retirees and dependents differed substantially in baseline characteristics. Retirees were older (median 50 versus 46 years, *p* = 0.003), far more likely to be male (52.0% versus 7.0%, *p* < 0.001), and more likely to be Hispanic (22.8% versus 11.4%, *p* = 0.004) or Black (12.2% versus 5.1%, *p* = 0.052). They exhibited a higher burden of hypertension (63.4% versus 38.0%, *p* < 0.001) and obstructive sleep apnea (80.5% versus 56.3%, *p* < 0.001), along with greater total number of comorbidities (median 5.0 versus 4.0 comorbidities, *p* < 0.001). Preoperative BMI was similar between groups (median 40.3 versus 39.7 kg/m^2^, *p* = 0.819). Retirees had higher hemoglobin and HbA1c than dependents. Lipid profiles were similar with exception of lower high-density lipoprotein in retirees (median 44.0 versus 47.0, *p* = 0.017).

**Table 1:** Demographics and preoperative characteristics stratified by beneficiary status

Variable	Overall	Dependent (*N* = 158)	Retiree (*N* = 123)	*p*-value
Demographics				
Age (years)	48.0 (41.0–55.0)	46.0 (38.0–54.0)	50.0 (43.0–56.0)	**0.003**
Female sex	206 (73.3)	147 (93.0)	59 (48.0)	** < 0.001**
White race	144 (51.3)	83 (52.5)	61 (49.6)	0.701
Black race	23 (8.2)	8 (5.1)	15 (12.2)	0.052
Hispanic ethnicity	46 (16.4)	18 (11.4)	28 (22.8)	0.017
Other race	68 (24.2)	49 (31.0)	19 (15.5)	**0.004**
Married	233 (82.9)	137 (86.7)	96 (78.1)	0.079
Home distance (miles)	23.0 (17.0–30.0)	23.0 (15.0–30.0)	22.0 (17.7–30.0)	0.909
Preoperative characteristics				
ASA class				0.410
1	2 (0.7)	2 (1.3)	0 (0.0)	
2	99 (35.2)	62 (39.2)	37 (30.1)	
3	178 (63.4)	93 (58.9)	85 (69.1)	
4	2 (0.7)	1 (0.6)	1 (0.8)	
Comorbidities				
Prediabetes	99 (35.2)	53 (33.5)	46 (37.4)	0.612
Type 2 diabetes mellitus	52 (18.5)	23 (14.6)	29 (23.6)	0.076
Obstructive sleep apnea	188 (66.9)	89 (56.3)	99 (80.5)	** < 0.001**
Hypertension	138 (49.1)	60 (38.0)	78 (63.4)	** < 0.001**
Hyperlipidemia	172 (61.2)	89 (56.3)	83 (67.5)	0.075
NAFLD	70 (24.9)	44 (27.9)	26 (21.1)	0.203
GERD	191 (68.0)	108 (68.4)	83 (67.5)	0.919
Atrial fibrillation	8 (2.9)	2 (1.3)	6 (4.9)	0.153
Chronic pain	89 (31.7)	46 (29.1)	43 (35.0)	0.403
Psychiatric diagnosis	172 (61.2)	95 (60.1)	77 (62.6)	0.810
Sum of comorbidities	4.0 (3.0–5.0)	4.0 (3.0–5.0)	5.0 (4.0–6.0)	** < 0.001**
Smoking history	78 (27.8)	37 (23.4)	41 (33.3)	0.088
Hiatal hernia	100 (35.6)	58 (36.7)	42 (34.2)	0.721
Medication/therapy				
PPI/H2 blocker use	144 (51.3)	83 (52.5)	61 (49.6)	0.710
Statin use	76 (27.1)	33 (20.9)	43 (35.0)	**0.012**
Sum of antihypertensives	0.0 (0.0–1.0)	0.0 (0.0–1.0)	1 (0.0–1.0)	**0.003**
CPAP adherence	120 (65.6)	53 (61.6)	67 (69.1)	0.423
Surgical history				0.512
LAGB	4 (8.5)	3 (9.1)	1 (7.1)	
LAGB converted to Nissen	1 (2.1)	0 (0.0)	1 (7.1)	
LAGB converted to SG	1 (2.1)	1 (3.0)	0 (0.0)	
LSG	39 (83.0)	28 (84.9)	11 (78.6)	
Nissen fundoplication	2 (4.3)	1 (3.0)	1 (7.1)	
Metabolic profiles				
Weight (kg)	111.1 (97.9–126.5)	103.9 (91.7–119.2)	119.6 (103.6–130.4)	** < 0.001**
Height (cm)	165.1 (160.0–172.7)	162.6 (157.5–165.1)	170.2 (162.6–177.8)	** < 0.001**
BMI (kg/m^2^)	40.0 (36.6–43.6)	39.7 (36.1–44.1)	40.3 (37.0–43.2)	0.819
Ideal body weight (kg)	68.2 (64.0–74.6)	66.1 (62.0– 68.2)	72.4 (66.1–79.0)	** < 0.001**
Excess body weight (kg)	40.85 (31.1–53.1)	38.5 (28.7–52.6)	44.9 (36.4–54.4)	**0.011**
Preoperative anemia	37 (13.4)	24 (15.5)	13 (10.7)	0.310
Hemoglobin (g/dL)	13.6 (12.8–14.6)	13.4 (12.5–14.3)	14.2 (13.0–15.1)	** < 0.001**
HbA1c (%)	5.7 (5.3–6.0)	5.5 (5.2–5.9)	5.8 (5.4–6.1)	**0.003**
Total cholesterol (mg/dL)	185.0 (157.0–213.0)	182.0 (153.0–208.0)	186.0 (160.0–216.0)	0.410
HDL (mg/dL)	45.5 (39.0–54.2)	47.0 (40.0–57.0)	44.0 (38.0–52.0)	**0.017**
LDL (mg/dL)	110.0 (86.0–134.0)	107.0 (85.0–132.0)	112.5 (87.5–136.5)	0.210
Triglycerides (mg/dL)	111.0 (82.0–170.0)	107.0 (77.0–163.0)	112.0 (88.0–176.0)	0.332

Bold values indicate statistical significance (*p* < 0.05)

*ASA* American Society of Anesthesiologists, *BMI* body mass index, *CPAP* continuous positive airway pressure, *GERD* gastroesophageal reflux disease, *H2* histamine, *HbA1c* hemoglobin A1c, *HDL* high-density lipoprotein, *LAGB* laparoscopic adjustable gastric banding, *LDL* low-density lipoprotein, *LSG* laparoscopic sleeve gastrectomy, *NAFLD* non-alcoholic fatty liver disease, *PPI* proton pump inhibitor. Categorical variables reported as frequency (percentage) and continuous variables reported as median (interquartile range)

#### Operative and perioperative characteristics

Table 2 summarizes the operative and perioperative characteristics between beneficiary groups. Operative approach and procedure type distributions were mostly similar between groups, though retirees underwent fewer revisional/conversion procedures compared with dependents (11.4% versus 20.9%, *p* = 0.049). Intraoperative complications were less than 4% overall and comparable between groups. Perioperative recovery metrics showed only modest differences. Retirees had a shorter median hospital length of stay (1 versus 2 days, *p* = 0.005). Perioperative opioid use, antiemetic requirements, early oral intake, and 24-h changes in hemoglobin were similar between groups.

**Table 2:** Operative characteristics and perioperative outcomes stratified by beneficiary status

Variable	Overall	Dependent (*N* = 158)	Retiree (*N* = 123)	*p*-value
Operative characteristics				
Laparoscopic approach	276 (98.2)	155 (98.1)	121 (98.4)	0.919
Robotic approach	5 (1.8)	3 (1.9)	2 (1.6)	0.921
Sleeve gastrectomy	124 (44.1)	63 (39.9)	61 (49.6)	0.133
Roux-en-Y gastric bypass	157 (55.9)	95 (60.1)	62 (50.4)	0.131
Revision/conversion procedure	47 (16.7)	33 (20.9)	14 (11.4)	**0.049**
Concurrent HH repair	116 (41.3)	71 (44.9)	45 (36.6)	0.210
Operative time (minutes)	135.0 (103.0–179.0)	134.5 (104.0–184.0)	137.0 (100.0–170.0)	0.321
Intraoperative complication	10 (3.6)	6 (3.8)	4 (3.3)	0.937
Perioperative outcomes				
Overnight antiemetic doses	0.0 (0.0–1.0)	1.0 (0.0–1.0)	0.0 (0.0–1.0)	0.201
Overnight oxycodone (mg)	5.0 (0.0–10.0)	5.0 (0.0–10.0)	5.0 (0.0–10.0)	0.812
Overnight hydromorphone (mg)	0.0 (0.0–0.2)	0.0 (0.0–0.2)	0.0 (0.0–0.3)	0.832
Overnight morphine equivalents (mg)	8.0 (2.0–16.0)	8.3 (1.0–15.0)	8.0 (2.0–16.0)	0.818
Overnight PO intake (mL)	150.0 (30.0–300.0)	122.5 (30.0–290.0)	150.0 (60.0–330.0)	0.110
POD1 PO intake (mL)	515.0 (290.0–780.0)	500.0 (260.0–760.0)	555.0 (300.0–850.0)	0.205
Oxycodone through POD1 (mg)	15.0 (5.0–25.0)	15.0 (5.0–25.0)	15.0 (5.0–25.0)	0.619
Hydromorphone through POD1 (mg)	0.4 (0.2–0.9)	0.4 (0.2–0.9)	0.4 (0.2–0.9)	0.703
Morphine equivalents through POD1 (mg)	23.5 (9.5–44.5)	24.5 (11.0–44.0)	23.5 (8.5–45.0)	0.613
POD1 hemoglobin decrease ≥ 2 (g/dL)	51 (18.2)	31 (19.6)	20 (16.3)	0.606
Length of stay (days)	1.0 (1.0–2.0)	2.0 (1.0–2.0)	1.0 (1.0–2.0)	**0.005**
Postoperative complication	14 (5.0)	10 (6.4)	4 (3.3)	0.401

Bold values indicate statistical significance (*p* < 0.05)

*HH* hiatal hernia, *POD* postoperative day. Categorical variables are reported as frequency (percentage) and continuous variables are reported as median (interquartile range)

#### Complications, healthcare utilization, and weight loss outcomes

Median follow-up was 30.1 months (22.8–37.7) overall, with similarly robust follow-up among dependents (28.8 months) and retirees (32.0 months). Availability of outcome data was excellent, with 100% of patients having at least one component of 12-month outcome data. Table 3 compiles the complication, readmission, and ED visit rates, laboratory results, weight loss outcomes, and cardiometabolic results at follow-up intervals. Twelve-month complication rates, including anastomotic stricture, marginal ulcer, obstruction, and surgical site infection rates, did not differ significantly between retirees and dependents. Readmissions and all-cause ED visits at 30 days, 6 months, and 12 months occurred at mostly similar frequencies in unadjusted analysis; there were fewer all-cause ED visits at 30 days (*p* = 0.027) and surgical ED visits at 12 months in retirees than dependents (*p* = 0.048). Weight loss outcomes, including percent TWL and percent EWL at 6, 12, and 24 months, were comparable. Weight at 6, 12, and 24 months was significantly higher in retirees compared to dependents, which reflected the sex imbalances between groups. Retirees experienced a greater reduction in HbA1c (median − 0.4 versus − 0.3, *p* = 0.020), but improvements in lipid profiles were comparable. Unadjusted differences motivated the use of causal-adjustment methods given the substantial baseline imbalance, particularly with respect to sex distribution.

**Table 3:** Postoperative outcomes at 12—24 months stratified by beneficiary status

Variable	Overall*	Dependent	Retiree	*p*-value
Healthcare utilization through 12 months				
Surgical complication, 12 months	40 (14.3)	23 (14.7)	17 (13.9)	0.901
Anastomotic stricture	5 (2.4)	4 (3.5)	1 (1.1)	0.532
Marginal ulcer	16 (7.7)	10 (8.8)	6 (6.5)	0.702
SSI/other infection	8 (3.9)	3 (2.7)	5 (5.3)	0.517
Small bowel obstruction	5 (2.5)	3 (2.7)	2 (2.2)	0.911
Other	12 (5.8)	6 (5.4)	6 (6.4)	0.913
Reoperation, 12 months	7 (2.5)	4 (2.5)	3 (2.4)	0.903
EGD for symptoms, 12 months	25 (8.9)	16 (10.1)	9 (7.3)	0.241
Readmission, 30 days	25 (9.0)	17 (10.8)	8 (6.6)	0.305
Readmission, 6 months	44 (15.8)	25 (15.9)	19 (15.6)	0.943
Readmission, 12 months	63 (22.6)	37 (23.6)	26 (21.3)	0.801
All-cause ED visit, 30 days	77 (27.6)	52 (33.1)	25 (20.5)	**0.027**
All-cause ED visit, 6 months	123 (44.1)	77 (49.0)	46 (37.7)	0.077
All-cause ED visit, 12 months	150 (53.8)	89 (56.7)	61 (50.0)	0.301
Surgical ED visit, 12 months	77 (27.4)	51 (32.3)	26 (21.1)	**0.048**
Laboratory results at 12 months				
Anemia	43 (17.3)	28 (19.4)	15 (14.3)	0.413
Hemoglobin (g/dL)	13.3 (12.5–14.1)	13.0 (12.3–13.9)	13.5 (12.7–14.4)	**0.002**
HbA1c (%)	5.3 (5.0–5.5)	5.2 (5.0–5.4)	5.3 (5.0–5.5)	**0.041**
HbA1c change	– 0.4 (– 0.7 to – 0.1)	– 0.3 (– 0.6 to 0.0)	– 0.4 (– 0.8 to – 0.2)	**0.020**
Total cholesterol (mg/dL)	163.0 (142.0–187.0)	160.0 (141.0–180.5)	167.0 (142.0–193.0)	0.208
Total cholesterol change (%)	– 10.8 (– 21.3–1.5)	– 11.3 (– 22.9–0.6)	– 10.3 (– 21.0–2.7)	0.609
HDL (mg/dL)	55.0 (46.0–62.0)	55.0 (48.5–62.0)	55.0 (44.0–62.0)	0.445
HDL change (%)	17.1 (2.4–33.3)	16.0 (2.3–31.6)	20.0 (2.4–36.4)	0.444
LDL (mg/dL)	89.0 (68.0–111.0)	85.0 (68.5–104.5)	92.0 (68.0–118.0)	0.238
LDL change (%)	– 18.4 (– 32.4–2.3)	– 21.2 (– 32.5–3.1)	– 15.9 (– 31.3–1.6)	0.513
Triglycerides (mg/dL)	84.0 (60.0–113.0)	81.5 (62.0–111.5)	88.0 (59.0–114.0)	0.648
Triglycerides change (%)	– 26.8 (– 46.2–7.6)	– 25.4 (– 46.0–7.8)	– 26.9 (– 46.7–7.4)	0.918
Weight loss outcomes through 24 months				
Weight, 6 months (kg)	84.8 (75.8–99.3)	79.9 (72.8–94.9)	92.4 (79.0–105.4)	**0.001**
BMI, 6 months (kg/m^2^)	30.7 (28.0–35.0)	30.6 (28.4–35.1)	30.7 (27.6–35.0)	0.933
TWL %, 6 months	22.0 (16.1–26.9)	22.0 (16.3–26.8)	22.0 (15.9–27.2)	0.734
EWL %, 6 months	59.0 (43.3–72.9)	59.4 (43.6–71.0)	58.7 (42.9–78.4)	0.601
Weight, 12 months (kg)	79.8 (69.3–95.2)	76.6 (68.6–87.4)	87.5 (73.4–99.4)	**0.001**
BMI, 12 months (kg/m^2^)	29.2 (26.0–33.0)	29.1 (26.1–32.2)	29.4 (25.9–33.5)	0.708
TWL %, 12 months	26.6 (21.4–33.1)	27.2 (21.9–32.7)	26.3 (20.7–33.4)	0.848
EWL %, 12 months	72.6 (54.5–92.0)	72.9 (55.8–90.0)	72.2 (51.5–92.5)	0.897
Weight, 24 months (kg)	80.9 (70.4–95.8)	76.8 (67.4–87.4)	86.2 (73.1–105.0)	**0.002**
BMI, 24 months (kg/m^2^)	29.5 (26.1–33.9)	29.3 (26.0–33.4)	29.7 (26.2–34.5)	0.589
TWL % at 24 months	27.2 (17.4–33.3)	26.8 (17.5–32.5)	27.5 (17.0–35.2)	0.898
EWL % at 24 months	72.1 (44.4–91.3)	75.9 (44.8–91.3)	66.8 (43.9–91.2)	0.695
Cardiometabolic outcomes at 12 months				
Stopped CPAP, 12 months	50 (49.0)	21 (50.0)	29 (48.3)	0.901
Statin de-escalation	62 (70.5)	32 (82.1)	30 (61.2)	0.058
Antihypertensive de-escalation	42 (51.9)	27 (65.9)	15 (37.5)	**0.020**
Protein intake ≥ 60 g/day	163 (76.2)	87 (71.3)	76 (82.6)	0.079
Aerobic exercise ≥ 150 min/week	120 (55.8)	66 (54.1)	54 (58.1)	0.700

Bold values indicate statistical significance (*p* < 0.05)

*BMI* body mass index, *CPAP* continuous positive airway pressure, *ED* emergency department, *EGD* esophagogastroduodenoscopy, *EWL* excess weight loss, *HbA1c* hemoglobin A1c, *HDL* high-density lipoprotein, *LDL* low-density lipoprotein, *SSI* skin/soft tissue infection, *TWL* total weight loss

^*^Denominators reflect the number of patients with available data at each follow-up interval. Healthcare utilization outcomes were available for N = 279—281 patients; laboratory outcomes at 12 months for N = 244—249 patients; weight-loss outcomes for approximately N = 270 at 6 months, N = 250 at 12 months, and N = 210 at 24 months. Cardiometabolic outcomes at 12 months varied by outcome eligibility (N = 81—215)

### Primary analysis: overlap-weighted regression

#### Covariate balance after overlap weighting

**Fig. 1:**
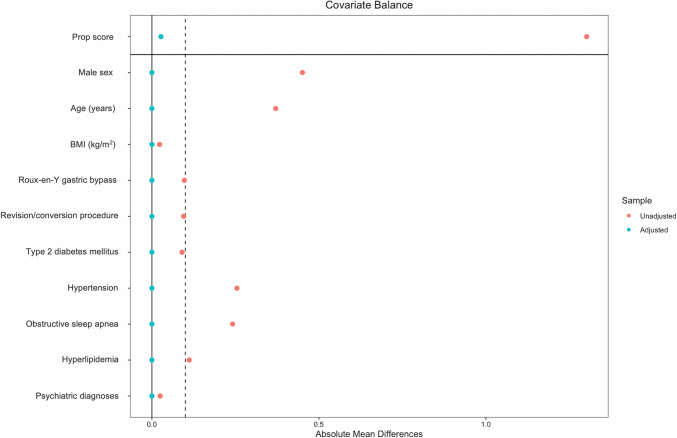
Covariate balance before and after overlap weighting. Love plot demonstrating standardized mean differences for key demographic and clinical covariates before and after overlap weighting. Substantial baseline imbalance between retirees and dependents was observed in unadjusted comparisons, particularly for sex, age, and comorbidity burden. After overlap weighting, all covariates achieved excellent balance, with standardized mean differences < 0.01, indicating near-complete elimination of measured confounding

Overlap weighting achieved exceptional covariate balance between retirees and dependents, reducing all standardized mean differences to < 0.01. The Love plot confirming this balance is provided as Fig. 1. Effective sample sizes were preserved, allowing full use of the cohort while minimizing confounding.

#### Adjusted perioperative and postoperative outcomes

**Fig. 2:**
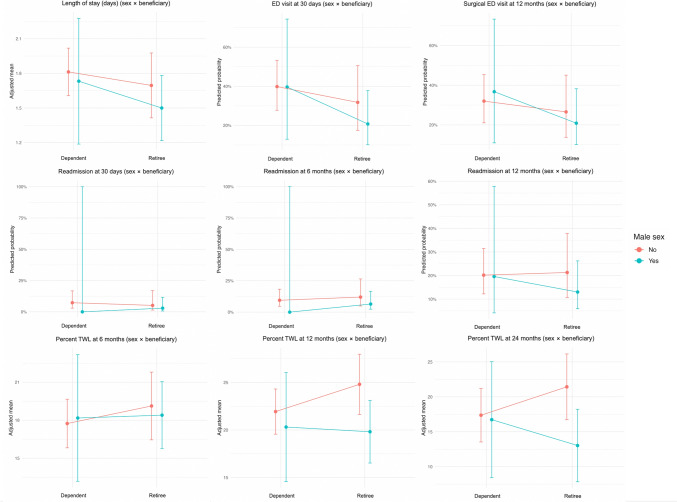
Marginal effects of beneficiary status and sex on adjusted outcomes. Marginal-effects plots showing adjusted predicted means or probabilities for length of stay, ED visits, readmissions, and percent total weight loss at 6, 12, and 24 months, stratified by beneficiary status and sex. Estimates were derived from overlap-weighted regression models including beneficiary status, sex, and their interaction term. Error bars represent 95% confidence intervals. No significant effect modification by sex was observed, and adjusted outcomes were similar between retirees and dependents across all time points

After weighting, beneficiary status was not independently associated with index hospital LOS. Moreover, beneficiary status did not predict ED visits (including surgical ED visits) or readmissions at 30 days, 6 months, or 12 months, or percent TWL at 6, 12, or 24 months. Point estimates were centered near unity with confidence intervals crossing 1. Marginal-effects plots (Fig. 2) confirmed similar adjusted predicted probabilities of ED visits, readmissions, and percent TWL for retirees and dependents across all timepoints. Overall, the overlap-weighted regression demonstrates that beneficiary status is not an independent determinant of hospital LOS, postoperative healthcare utilization, or short-term weight loss (Table 4A).

**Table 4:** Multivariable regression models for postoperative outcomes

Model	Outcome	OR	95% CI	*p*-value
4A. Overlap-weighted cohort				
Logistic				
	All-cause ED visit, 30 days	0.67	0.35–1.29	0.229
	Surgical ED visit, 12 months	0.78	0.41–1.49	0.443
	Readmission, 30 days	0.81	0.30–2.22	0.686
	Readmission, 6 months	1.38	0.66–2.88	0.396
	Readmission, 12 months	0.98	0.50–1.92	0.957
Linear				
	Hospital LOS, days	− 0.13	− 0.36–0.10	0.249
	Percent TWL, 6 months	1.28	− 1.18–3.75	0.307
	Percent TWL, 12 months	2.29	− 0.82–5.40	0.149
	Percent TWL, 24 months	3.53	− 0.71–7.77	0.102
4B. Female-only propensity-matched cohort				
Logistic				
	All-cause ED visit, 30 days	0.64	0.32–1.26	0.200
	Surgical ED visit, 12 months	0.71	0.35–1.41	0.329
	Readmission, 30 days	0.80	0.27–2.15	0.662
	Readmission, 6 months	1.49	0.66–3.29	0.330
	Readmission, 12 months	0.99	0.49–2.00	0.984
Linear				
	Hospital LOS, days	− 0.14	− 0.38–0.10	0.259
	Percent TWL, 6 months	1.16	− 1.11–3.44	0.314
	Percent TWL, 12 months	2.21	− 0.47–4.88	0.105
	Percent TWL, 24 months	2.47	− 1.46–6.40	0.216

*ED* emergency department, *LOS* length of stay, *TWL* total weight loss

### Effect modification by sex

Because sex distribution differed markedly between groups, interaction terms between beneficiary status (retiree) and sex (male) were incorporated into adjusted models. These analyses demonstrated no significant interaction for any perioperative, healthcare utilization, or weight loss outcome (Table S1). Adjusted predicted probabilities varied minimally across sex strata, indicating that the relationship between beneficiary status and these outcomes did not differ between men and women. In other words, any unadjusted differences are driven by sex imbalance rather than true differential effects of beneficiary status.

### Sensitivity analysis: female-only propensity score matching

#### Covariate balance in the matched cohort

**Fig. 3:**
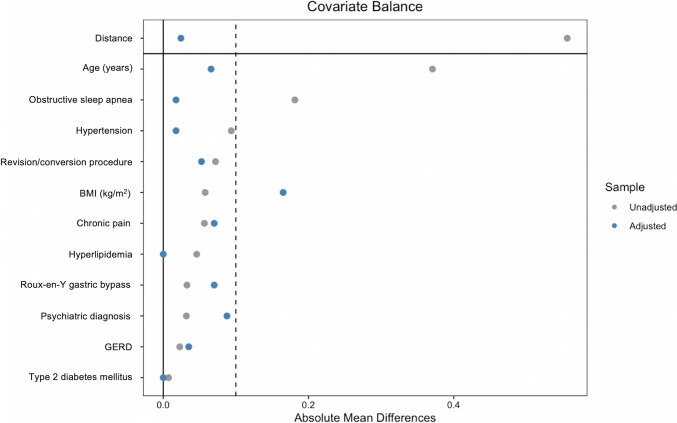
Covariate balance after female-only propensity score matching. Love plot demonstrating covariate balance in the female-only propensity score-matched cohort. Nearest-neighbor 1:1 matching without replacement was performed using a caliper of 0.2 standard deviations of the logit propensity score. After matching, all included covariates achieved standardized mean differences < 0.1, indicating adequate balance between female retirees and female dependents for sensitivity analyses

Propensity score matching yielded 57 female retiree–dependent pairs (*N* = 114). Post-matching covariate balance was excellent, with all standardized mean differences < 0.1. The matched cohort Love plot is provided in Fig. 3.

#### Matched perioperative and postoperative outcomes

After matching, retirees and dependents showed nearly identical demographic and preoperative clinical characteristics, confirming that matching effectively removed baseline imbalance. In the matched cohort, hospital LOS, ED visits (including surgical ED visits), and readmissions at 30 days, 6 months, and 12 months did not differ between groups. Percent TWL was also similar at all time points. The female-only propensity score matching corroborated the findings from the overlap-weighted models and confirmed that beneficiary status does not influence these outcomes when sex as a confounder is adequately controlled (Table 4B).

## Discussion

In this single-center retrospective analysis, beneficiary status was not independently associated with perioperative outcomes, postoperative healthcare utilization, or short-term weight loss after bariatric surgery. Although retirees differed substantially from dependents at baseline, particularly with respect to age, sex distribution, and comorbidity burden, these differences did not translate into adjusted differences in LOS, ED utilization, readmissions, or percent TWL once confounding was addressed. Using overlap-weighted regression as the primary analytic approach, and female-only propensity score matching as a sensitivity analysis, we demonstrate that beneficiary status itself does not appear to be a determinant of bariatric surgery outcomes in the direct-care MTF setting.

Our findings extend the limited prior literature evaluating bariatric surgery outcomes among MHS beneficiaries. Authors from our institution, Roe et al., [10] compared retirees and dependents and found less short-term postoperative weight loss among retirees, however, these analyses were limited by small sample size and reliance on largely unadjusted comparisons. Caponera et al. [11] examined bariatric surgery outcomes among military beneficiaries with a focus on diabetes remission, but did not specifically evaluate retiree-dependent differences in weight loss or postoperative healthcare utilization, nor did they address the substantial demographic imbalance between beneficiary categories. A critical limitation of prior studies is the lack of explicit adjustment for sex, despite well-established associations between sex, baseline body composition, comorbidity burden, and postoperative weight loss trajectories [11]. In contrast, our analysis leveraged a contemporary cohort and applied propensity-based and overlap-weighted models designed to account for clinical imbalance between retirees and dependents, particularly the marked sex disparity. After adjustment, beneficiary status was not independently associated with weight loss outcomes, contrary to existing, albeit limited, literature. More broadly, our results align with national descriptions of bariatric surgery within the MHS. Prior analyses across all MTFs have demonstrated that bariatric case volume, procedure mix, and complication rates are comparable to large civilian registries, and that dependents of retirees comprise the largest beneficiary subgroup undergoing bariatric surgery [5, 14]. Our study builds on this foundation by directly testing whether beneficiary category independently predicts outcomes, and suggests that once access to surgery is achieved within a standardized MTF program, outcomes are similar across beneficiary groups.

Although retirees were statistically older than dependents, the absolute difference in median age was modest (50 vs. 46 years), and the age distributions between groups were broad and overlapping. Both cohorts spanned multiple decades of life, with meaningful representation across age strata rather than clustering within narrow age bands. Prior Military Health System studies have demonstrated similar degrees of age separation between beneficiary groups [10, 11], suggesting that this pattern reflects the expected demographic structure rather than an artifact of cohort selection. Therefore, it is unlikely that the lack of observed differences in outcomes is attributable to excessive homogeneity between groups, but rather reflects true clinical comparability after adjustment for baseline characteristics.

From a clinical and policy perspective, these findings are reassuring. Bariatric surgery is increasingly recognized as a high-value intervention for reducing long-term cardiometabolic risk and downstream healthcare utilization [6, 15]. Our data suggest that retiree status alone should not be viewed as a risk factor for inferior perioperative outcomes or diminished weight loss response when patients are treated within a standardized MTF bariatric program. This has implications for patient counseling, surgical candidacy decisions, and resource planning, particularly as the retiree population within the MHS continues to grow.

Several limitations warrant consideration. This was a single-center study conducted within a direct-care MTF, and results may not generalize to purchased-care settings or civilian hospitals serving TRICARE beneficiaries. Again, as a retrospective analysis with a fixed-cohort size, an a priori sample size calculation was not performed. A post hoc detectable-difference assessment demonstrated that, with 158 dependents and 123 retirees, the study had 80% power (two-sided *α* = 0.05) to detect absolute differences of approximately 16 percentage points for 12-month all-cause ED utilization and 15.6 percentage points for 12-month readmissions. Thus, while large clinically meaningful differences in postoperative healthcare utilization are unlikely within this cohort, smaller magnitude differences cannot be excluded, which is a limitation of our sample size. Although follow-up was robust for 12-month outcomes, attrition increased at later time points, which may introduce bias in 24-month weight loss estimates. Residual confounding is possible, particularly with respect to socioeconomic variables and psychosocial factors not fully captured in our dataset. Lastly, postoperative healthcare utilization outside the MHS network may have been incompletely captured, potentially leading to underestimation of ED visits or hospitalizations among beneficiaries who sought care in civilian facilities.

In conclusion, beneficiary status was not independently associated with perioperative outcomes, postoperative healthcare utilization, or short-term weight loss after bariatric surgery when demographic and clinical differences were accounted for. Apparent unadjusted differences between retirees and dependents were largely explained by underlying patient characteristics, particularly sex distribution. The findings from our single-center analysis suggest that bariatric surgery outcomes within the MHS are equitable across beneficiary categories. Multicenter studies are warranted to confirm these results and to further evaluate the influence of beneficiary status across diverse practice settings.

## Supplementary Information

Below is the link to the electronic supplementary material.

Supplementary file1 (DOCX 13 KB)
