# Supplementary material for: Impact of beneficiary status on bariatric surgery outcomes in a military treatment facility
Source: Surg Endosc. 2026 May 18;40(6):5347–57. doi: 10.1007/s00464-026-12860-z (PMC13246537; doi:10.1007/s00464-026-12860-z)
Supplement: Supplementary file 1 — Supplementary file1 (DOCX 13 KB) [file 464_2026_12860_MOESM1_ESM.docx]

| **Table S1. Interaction p-values: retiree × male sex** | | |
| --- | --- | --- |
| **Model** | **Outcome** | **Interaction**  ***p*-value** |
| **Logistic regression** | | |
|  | All-cause ED visits, 30 days | 0.530 |
|  | Readmission, 30 days | 0.989 |
|  | Readmission, 6 months | 0.988 |
|  | Readmission, 12 months | 0.577 |
|  | Surgical ED visits, 12 months | 0.572 |
| **Linear regression** | | |
|  | Hospital LOS, days | 0.716 |
|  | Percent TWL, 6 months | 0.691 |
|  | Percent TWL, 12 months | 0.319 |
|  | Percent TWL, 24 months | 0.124 |
| Abbreviations: ED, emergency department; LOS, length of stay; TWL, total weight loss | | |
